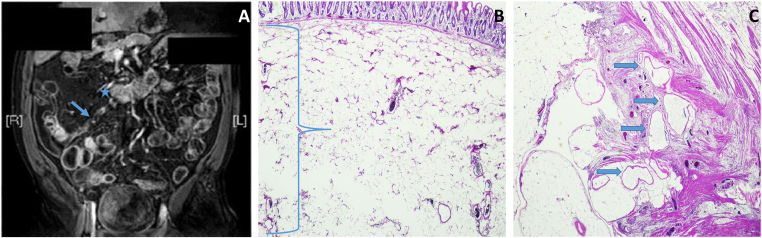# Ileal Lipohyperplasia Masquerading as Stricturing Crohn’s Disease

**DOI:** 10.1016/j.gastha.2022.11.009

**Published:** 2022-11-15

**Authors:** Muhammad B. Hammami, Nathalie H. Urrunaga

**Affiliations:** Division of Gastroenterology and Hepatology, University of Maryland School of Medicine, Baltimore, Maryland

A 47-year-old male with stricturing Crohn’s disease (CD) presented with intermittent right-lower-quadrant abdominal pain (RLQAP), nausea/vomiting, and constipation.

His history revealed small bowel resections (age 20/32 years) followed by 9 years of symptomatic remission with sulfasalazine, which was discontinued 7 years ago (medical insurance lapse). For 6 years, he had mild, intermittent, self-resolving RLQAP with nausea/vomiting that became more frequent two months ago, necessitating two hospitalizations (unremarkable esophagogastroduodenoscopy, colonoscopy, barium enema).

On presentation, he had mild RLQAP and tenderness without rebound/distention. Magnetic resonance enterography showed a 4-cm-dilated distal ileum (Figure A, asterisk), with 3 × 0.6-cm stricture proximal to the ileocecal valve (Figure A, arrow). Ileocecectomy revealed prominent adipose tissue in the submucosa between the mucosa and muscularis propria (Figure B, right brace) and dilated lymphatic vessels in the terminal ileum (Figure C, arrows) without evidence of CD.

This is the first case of lipohyperplasia causing small bowel stricture/obstruction in CD. Although management is still resection, lipohyperplasia should be considered in the differential diagnosis of small bowel stricture even in patients with known stricturing CD.

**Figure:**